# Predictive factors for recurrence after lower limb deformity correction in hypophosphatemic rickets

**DOI:** 10.1186/s13018-023-03963-7

**Published:** 2023-07-07

**Authors:** Chayut Suparatchatadej, Nath Adulkasem, Thanase Ariyawatkul, Perajit Eamsobhana, Chatupon Chotigavanichaya, Jidapa Wongcharoenwatana

**Affiliations:** Department of Orthopaedic Surgery, Faculty of Medicine Siriraj Hospital, Mahidol University, 2 Prannok Road, Bangkoknoi, 10700 Bangkok Thailand

**Keywords:** Rickets, Predictive, Recurrence, Deformity correction, Lower limbs

## Abstract

**Background:**

Surgical treatment for severe lower limb deformities in patients with hypophosphatemic rickets has shown satisfactory outcomes. However, the rates of recurrence of deformities after surgical correction were high, and studies on predictive factors of recurrence were limited. This study aimed to determine the predictive factors for the recurrence of lower limb deformities after surgical correction in patients with hypophosphatemic rickets, and the effects of each predictor on the recurrence of deformities.

**Methods:**

We retrospectively reviewed the medical records of 16 patients with hypophosphatemic rickets aged 5–20 years and who had undergone corrective osteotomies between January 2005 and March 2019. Demographic data from the patients, biochemical profiles, and radiographic parameters were collected. Univariable Cox proportional hazard analyses of recurrence were performed. Kaplan–Meier failure estimation curves for deformity recurrences of potential predictors were created.

**Results:**

A total of 38 bone segments were divided into 2 groups: 8 segments with recurrent deformities and 30 segments without recurrent. The average follow-up time was 5.5 ± 4.6 years. Univariable Cox proportional hazard analyses of recurrence found that an age < 10 years (hazard ratio [HR], 5.5; 95% CI, 1.1–27.1; *p* = 0.04), and gradual correction by hemiepiphysiodesis (HR, 7.0; 95% CI, 1.2–42.7; *p* = 0.03) were associated with recurrence after surgery. The Kaplan–Meier failure estimation for deformity recurrences by age at the time of surgery also achieved a statistically significant difference between ages < 10 years and those > 10 years (*p* = 0.02).

**Conclusions:**

Identifying predictive factors for the recurrence of lower limb deformities after surgical correction in hypophosphatemic rickets can assist in early recognition, proper intervention, and prevention. We found that an age < 10 years at the time of surgery was associated with recurrence after deformity correction and gradual correction with hemiepiphysiodesis may also be a potential factor affecting the recurrence.

## Introduction

Hypophosphatemic rickets is a disease caused by bone and cartilage mineralization defects secondary to renal phosphate wasting [1]. Patient typically presents with delayed linear growth and deformities of the lower limbs, commonly around the knee. The deformities include bilateral genu varum or valgum, bowing of the femur or tibia, torsional deformity of the tibia, and windswept deformity. These defects are usually noticeable after 1 or 2 years of age [2–4]. Typical radiological findings are widened and irregular physes, cupped and flared metaphyses, and generalized osteopenia [2]. The biochemical profile mainly consists of hypophosphatemia, hyperphosphaturia, decreased or slightly decreased 1,25-dihydroxy vitamin D levels, and increased alkaline phosphatase levels. However, calcium and parathyroid hormone (PTH) levels are frequently normal at presentation [2–5].

The conventional treatment to reverse and prevent further deformities is pharmacological by supplementation with phosphate and vitamin D analog [3, 6]. Surgical treatment may be required if the patient has severe or progressive deformities despite optimal pharmacological treatment [3]. Different surgical techniques, including corrective osteotomy and external fixation or intramedullary nailing, can effectively restore neutral lower limb alignment [7]. Nevertheless, previous studies reported that the recurrence rate of deformities remained as high as 90% during the follow-up period [2, 3, 7, 8].

The establishment of predictive factors will benefit patients with hypophosphatemic rickets through early prevention, early detection, and appropriate management of recurrent deformities. Several factors have been associated with recurrence, such as an osteotomy during childhood [3, 8]. However, there are limited studies on the predictive factors. This study aimed to identify factors predicting the recurrence of deformities after surgical correction in hypophosphatemic rickets and the effects of each factor on the recurrence of deformities.

## Methods

### Study design and patient evaluation

Following institutional review board approval, we retrospectively reviewed the registry of our hypophosphatemic rickets patients. Inclusion criteria were hypophosphatemic rickets patients with severe lower limb deformities who underwent deformity correction surgery between January 2005 and March 2019 with preoperative and postoperative radiographs. Patients were identified using code E83.3 (disorders of phosphorus metabolism and phosphatases) of the International Classification of Diseases, Tenth Revision (ICD-10). We excluded patients whose deformities developed due to other causes, such as post-traumatic complications and congenital deformities, and with incomplete clinical and radiographic data.

According to clinical practice recommendations [9], all patients were evaluated by a pediatric endocrinologist who confirmed the diagnosis based on clinical and biochemical profiles, and radiographical findings. After the diagnosis had been established, treatment started according to a standard protocol, including oral supplementation with calcium, phosphate, and vitamin D. The medical treatment aimed to control biochemical profiles by laboratory tests. All of our patients received appropriate medical treatment before surgery and maintained throughout growth. Patients and their parents were educated concerning the course of treatment, prognosis, long-term goals, and the consequences of noncompliance that would result in recurrence.

**Fig. 1:**
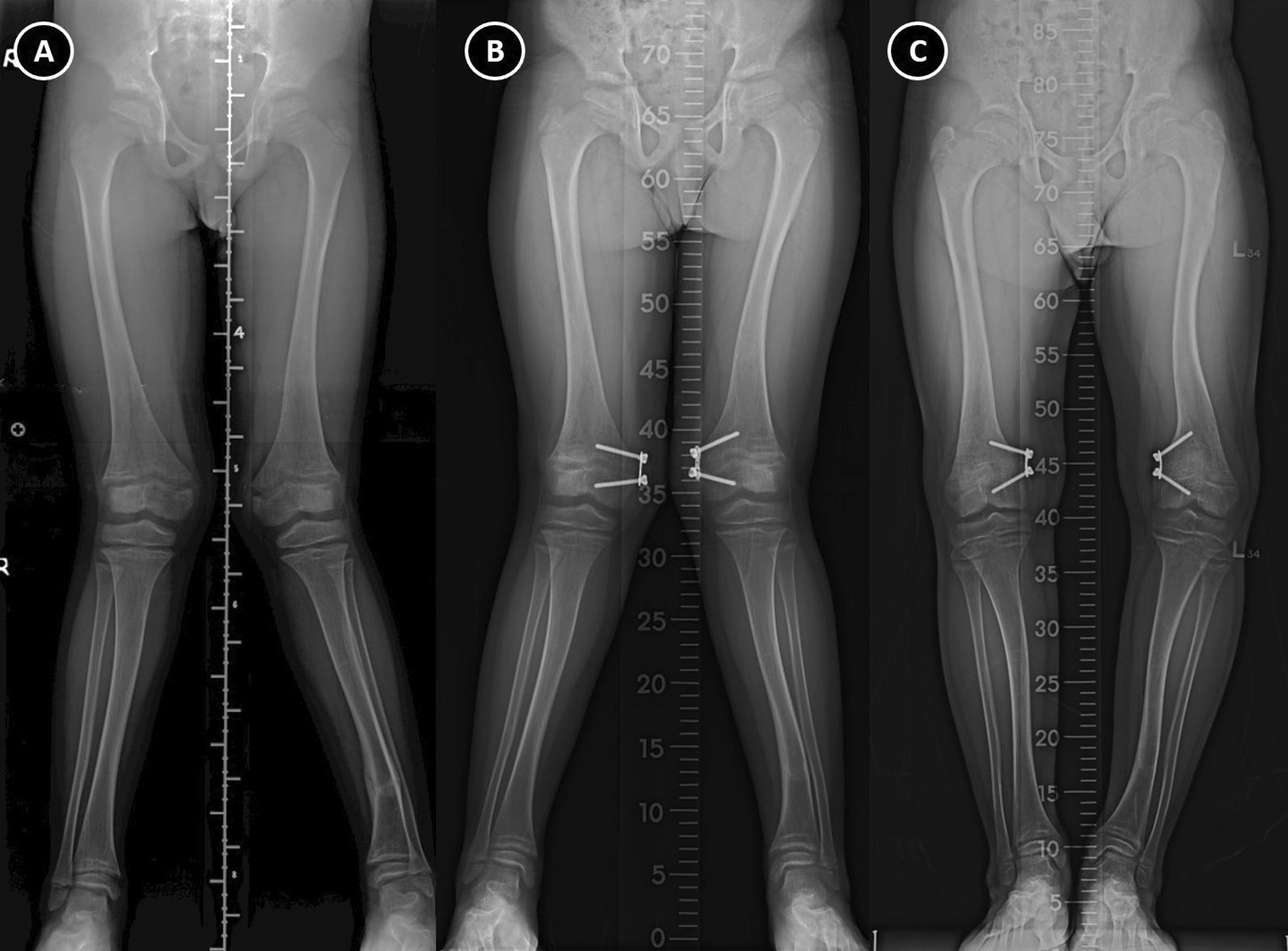
A 9-year-old male with hypophosphatemic rickets presented with bilateral genu valgum (**A**). Growth modulation performed at 10.2 years at bilateral distal femur (**B**). Latest radiographs before implant removal demonstrating overcorrection into varus bilaterally (**C**)

The indications for surgery included gait disturbance, medial or lateral thrust knee or instability, and pain. Surgical corrections were planned according to clinical assessment and the extent of the deformity. The selection of surgical correction methods and fixation techniques was according to the patient’s age, maturity of the growth plate, bone quality, and degrees of deformity that needed to be corrected. We corrected the deformities back to anatomic or near anatomic as possible. Radiographs of the knee joint and a full-length standing AP view of both lower extremities including the hip, knee, and ankle were assessed preoperatively, postoperatively, and at each follow-up (Fig. 1).

### Study variables and candidate predictors

**Fig. 2:**
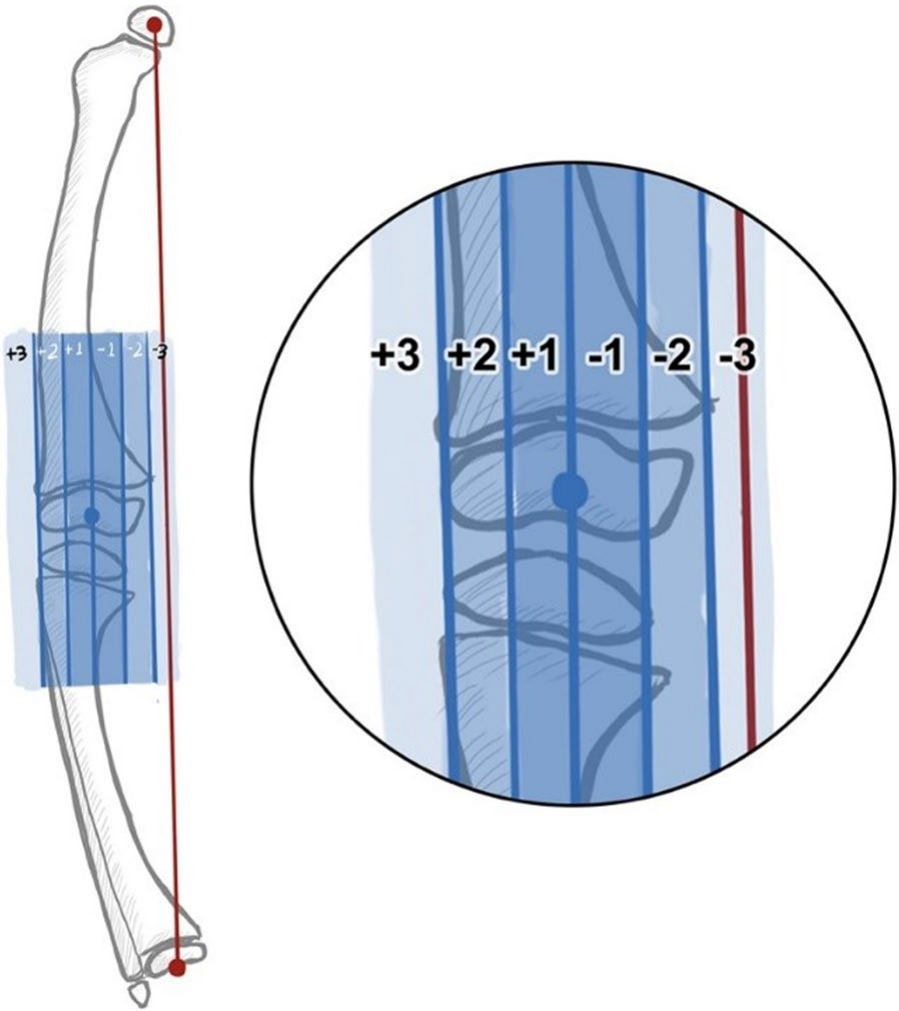
This figure shows mechanical axis zones of the lower limb (adapted from Stevens^11^): Zone − 1 to + 1 (neutral), Zone − 2 to − 3 (varus), Zone + 2 to + 3 (valgus)

Demographic data of the patients (age, sex, laterality, bone segments) and preoperative biochemical profiles such as serum calcium, phosphate, vitamin D, PTH, and alkaline phosphatase were collected. The preoperative radiographic parameters were measured, including mechanical axis deviation (MAD) and zones, femorotibial angle (FTA), mechanical lateral distal femoral angle (mLDFA), lateral distal tibial angle (LDTA), medial proximal tibial angle (MPTA), and joint-line congruence angle (JLCA) [10, 11] (Fig. 2). Two trained investigators independently measured all radiographic measurements. The third investigator resolved any discrepancies. The radiographic parameters were measured using SiPACS software (Sikraft Solutions Ltd., Dhaka, Bangladesh).

### Clinical endpoints

All patients were monitored until reached skeletal maturity. We defined “Recurrence” as an increased MAD compared with immediate postoperative value and beyond the normal range or progressive postoperative deformity requiring surgical correction before skeletal maturity. Therefore, one patient could have more than one recurrence event during the follow-up period. The recurrence time was measured from the date of the latest surgery to each recurrence event or censoring date.

### Statistical analysis

#### Fundamental analysis

The data distribution was determined using histogram analyses and Shapiro–Wilk tests. Normally distributed continuous data were tested with an independent t-test and are presented as mean and standard deviation. In contrast, non-normally distributed data were tested with the Mann–Whitney *U* test and are presented as the median and interquartile range (IQR). Finally, categorical data were tested with Fisher’s exact probability test and are presented as counts and percentages.

#### Survival analysis of recurrence

The second analytical stage involved plotting Kaplan–Meier failure estimation curves for the recurrence of the deformity using the log-rank test for each potential predictor. Multivariable Cox proportional hazard analyses (variables with a *p*-value < 0.1 in the univariable analysis were included in the multivariable analysis) and Prentice, Williams, and Petersen gap time (PWP-GT) modeling were used to account for multiple recurrence events within the same patient [12]. The PWP-GT model allows investigators to determine the effect of each predictor according to event order. All statistical analyses were conducted with STATA 14 (StataCorp LLC, College Station, TX, USA). The level of statistical significance was set at a probability (*P*) value of < 0.05.

## Results

### Demographic data

A total of 16 patients (5 male and 11 female) with 38 bone segments (20 femurs and 18 tibias) were divided into 2 groups. The recurrence group had 8 (21.1%) bone segments, while the non-recurrence group had 30 (78.9%) segments. The total average age was 12.7 ± 9.1 years old and the average follow-up time was 5.5 ± 4.6 years. Most of the patients were diagnosed with X-linked hypophosphatemic rickets (89.5%). The most frequently missing data item was the vitamin D level. Only 13 (34.2%) data points for this parameter were obtained, consisting of 1 (7.7%) bone segment with recurrent deformities and 12 (92.3%) segments without recurrent deformities. There were no significant differences in subtypes of hypophosphatemic rickets and biochemical parameters between the recurrence and non-recurrence groups. Details of the other demographic and biochemical profiles were listed in Table 1.

**Table 1:** Characteristic of ricket patients with recurrent deformity

Characteristic	Recurrence (*n* = 8 segments)		No recurrence (*n* = 30 segments)		*p*-value
	Mean	SD	Mean	SD	
Demographic					
Age (years)	9.6	2.9	13.0	4.4	0.05
Gender (*n*, %)					
Male	3	37.5	9	30.0	
Female	5	62.5	21	70.0	0.69
Laterality (*n*, %)					
Right	4	50.0	16	53.3	
Left	4	50.0	14	46.7	1.00
Segment (*n*, %)					
Femur	5	62.5	15	50	
Tibia	3	37.5	15	50	0.53
Follow-up time (years) (median, IQR)	8.1	3.6, 11.2	3.8	2.3, 5.4	0.19
Biochemical profiles					
Ca_2_^a^ (mg/dL) [normal 8.8–10.2 mg/dL]	9.1	0.5	9.0	0.4	0.65
PO_4_^b^ (mg/dL) [normal 2.5–4.5 mg/dL]	2.6	0.4	2.4	0.9	0.49
Vit D^c^ (ng/mL) [normal 20–50 ng/mL]	28.4	–	26.3	11.1	–
PTH^d^ (pg/mL) [normal 15–65 pg/mL]	47.2	31.3	81.1	42.0	0.07
ALP^e^ (IU/L) [normal 39–117 IU/L]	453.7	64.6	439.0	242.4	0.88
Types of rickets (*n*, %)					
X-linked hypophosphatemic rickets	7	87.5	27	90.0	
Tumor-induced rickets	0	0.0	1	3.3	
AD^f^ hypophosphatemic rickets	1	12.5	2	6.7	0.63
Operative treatment (*n*, %)					
Acute correction with plate and screws	2	25.0	16	53.5	
Acute correction with circular frames	5	62.5	12	40.0	
Gradual correction with hemiepiphysiodesis	1	12.5	2	6.7	0.31

^a^Ca_2_, Calcium level

^b^PO_4_, Phosphate level

^c^Vit D, 25OH vitamin D

^d^PTH, Parathyroid hormone

^e^ALP, Alkaline phosphatase

^f^AD, Autosomal Dominant

*p < 0.05 was considered significant

### Radiographic parameters

Twenty-three limbs demonstrated varus deformity. Of which, the average MAD was − 4.5 ± 2.4 cm medial to the center of the knee in the recurrence group, compared to − 6.7 ± 2.5 cm in the non-recurrence group (*p* = 0.12). The average mLDFA, MPTA, and JLCA also showed no statistically significant difference between both groups (Table 2).

**Table 2:** Radiographic parameters of the deformity

Characteristic	Recurrence		No recurrence		*p*-value
	Mean	SD	Mean	SD	
Varus deformity (*n* = 23, 60.5%)					
Femoral-tibial angle (°)	− 22.8	5.1	− 30.3	15.8	0.31
Joint line convergence angle (°)	− 2.6	3.8	− 3.0	2.2	0.77
Lateral distal femoral angle (°)	107.3	3.6	107.2	9.0	0.99
Medial proximal tibial angle (°)	88.4	10.5	80.8	9.0	0.12
Lateral distal tibial angle (°)	89.8	2.8	95.4	10.7	0.27
Mechanical axis deviation (cm)	− 4.5	2.4	− 6.7	2.5	0.12
Valgus deformity (*n* = 15, 39.5%)					
Femoral-tibial angle (°)	+ 33.7	15.9	+ 28.6	14.9	0.63
Joint line convergence angle (°)	+ 3.3	2.9	+ 2.9	1.8	0.75
Lateral distal femoral angle (°)	72.0	4.6	74.4	8.1	0.65
Medial proximal tibial angle (°)	96.0	8.9	94.8	5.4	0.78
Lateral distal tibial angle (°)	94.0	4.0	90.3	4.9	0.27
Mechanical axis deviation (cm)	+ 5.9	3.3	+ 5.1	3.9	0.79

Mechanical axis deviation data presented (+) = lateral to midline, and (−) = medial to midline

*p < 0.05 was considered significant

Valgus deformity was observed in 15 limbs, with an average MAD of + 5.9 ± 3.3 cm lateral to the center of the knee in the recurrence group and + 5.1 ± 3.9 cm in non-recurrence (*p* = 0.79). The average mLDFA, MPTA, and JLCA also showed no statistically significant difference between both groups (Table 2).

### Recurrence

The recurrence time was chosen as a variable for both Cox proportional hazard analyses and the Kaplan–Meier failure estimation curve for the recurrence of deformities.

Univariable Cox proportional hazard analyses of recurrence under PWP-GT modeling identified 2 significant predictors: An age < 10 years at the time of surgery and corrective deformity with gradual correction by hemiepiphysiodesis. An age < 10 years old at the time of surgery significantly increased the adjusted hazard ratio by 5.5 times (age < 10 years; 95% CI, 1.1–27.1; *p* = 0.04), while gradual correction by hemiepiphysiodesis significantly increased the adjusted hazard ratio by 7.0 time (95% CI, 1.2–42.7; *p* = 0.03) (Table 3).

**Table 3:** Univariable Cox’s proportional hazard analysis of the recurrence under PWP-GT modeling

Predictors	uHR	95% CI		*p*-value
Age < 10 years	5.5	1.1	27.1	0.04*
Male	1.2	0.4	4.0	0.78
Varus alignment	2.3	0.6	8.5	0.22
Biochemical profiles				
Ca^a^ < 9 mg/dL	0.3	0.1	1.8	0.20
Phosphate < 2.5 mg/dL	0.8	0.2	3.3	0.73
PTH^b^ ≥ 65 pg/mL	1.2	0.2	9.8	0.84
ALP^c^ ≥ 360 IU/L	5.7	0.8	38.5	0.08
Operative treatment				
Acute correction with internal fixation	Ref			
Acute correction with external fixation	3.1	0.7	13.6	0.13
Gradual correction with hemiepiphysiodesis	7.0	1.2	42.7	0.03*

^a^Ca, Calcium level

^b^PTH, Parathyroid hormone

^c^ALP, Alkaline phosphatase

*p < 0.05 was considered significant

There were 3 cases treated with gradual correction with hemiepiphysiodesis. All of them were distal femoral valgus deformities. One of three cases recurred to severe valgus deformity (MAD = 5.31 cm, Zone +3) at approximately 9 months after 12 months of correction. Detail on comparing each treatment method is shown in Table 4.

**Table 4:** Comparison of characteristic parameters between each operation

Characteristic	Acute correction with internal fixation		Acute correction with external fixation		Gradual correction with hemiepiphysiodesis		*p*-value
	Mean	SD	Mean	SD	Mean	SD	
Age at initial surgery	13.9	4.9	10.9	3.6	10.7	1.2	0.09
Bone segments (*n*, %)							
Femur	9	45	8	40	3	15	0.23
Tibia	9	50	9	50	0	0	
Deformity (*n*, %)							
Varus	11	47.8	12	52.2	0	0	0.07
Valgus	7	46.7	5	33.3	3	20	
Severity							
MAD (cm)	5.2	3.6	4.2	3.3	4.0	1.6	0.67

MAD = Mechanical axis deviation

*p < 0.05 was considered significant

**Fig. 3:**
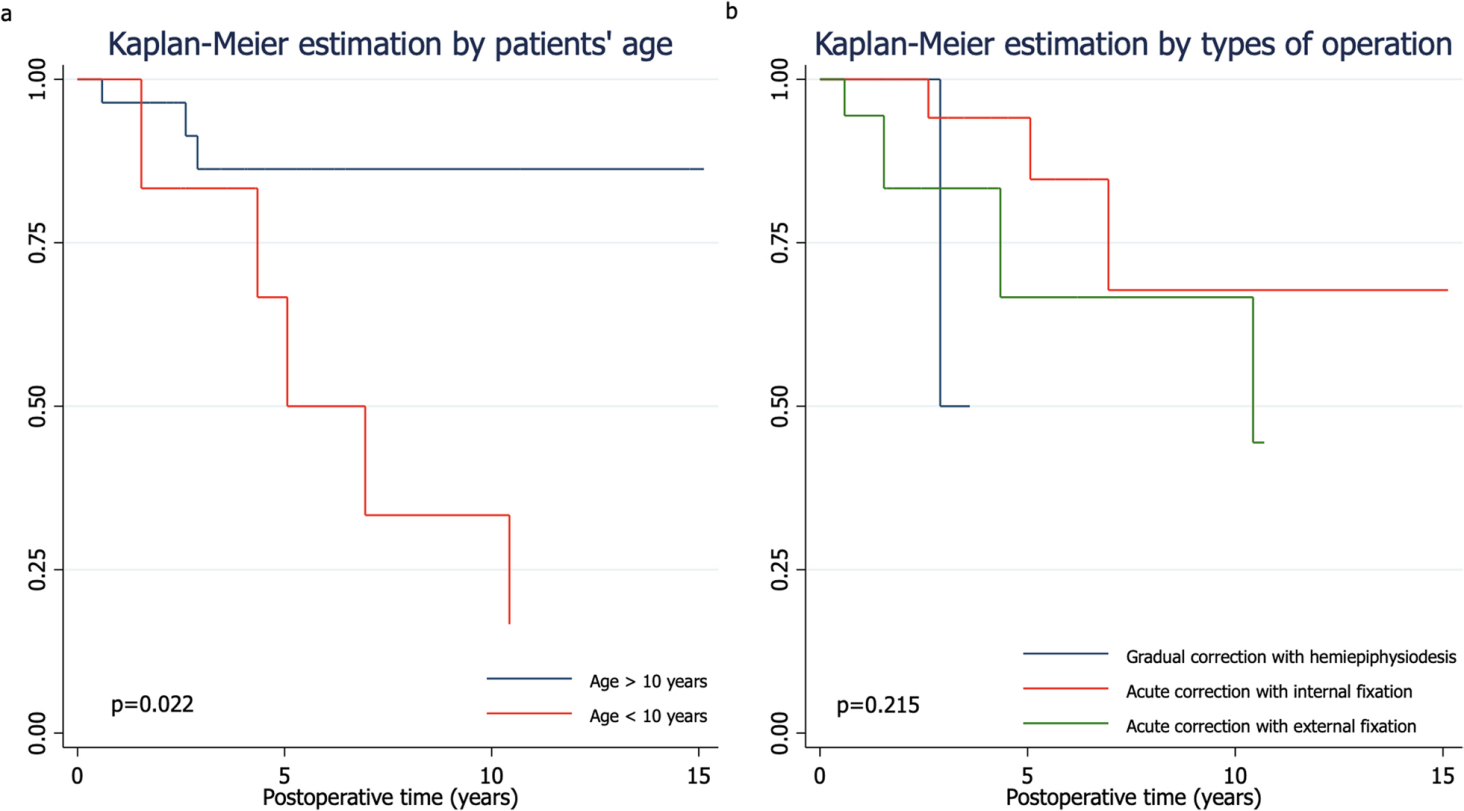
This graph shows Kaplan–Meier failure estimation curve for deformity recurrence with the log-rank test by **a** age at the time of surgery (*p* = 0.022), **b** type of operation (*p* = 0.215)

The Kaplan–Meier failure estimation curves for recurrent deformity using the log-rank test for each potential predictor found that the patient’s age had a significant difference in recurrence. At the time of surgery, an age < 10 years had a significantly higher deformity recurrence than an age > 10 years (*p* = 0.022). The analyses of the other potential parameters are illustrated in Fig. 3.

### Complications

From 38 bone segments, there were 2 cases of superficial pin tract infection, 2 cases of nonunion, 1 case of nerve injury, and 1 case of acute osteomyelitis. All patients received proper standard treatment with complete recovery.

## Discussion

Although surgical correction can rapidly correct residual skeletal deformities after medical treatment, several significant postoperative complications are a concern [3, 7]. Recurrence of deformity is one of the most common complications, ranging from 25 to 90% in hypophosphatemic rickets patients [3, 7], it leads to multiple surgeries of correction and removal of implants [13]. Our current study also found 21.1% of recurrence with age less than 10 years old at the time of surgery, and a gradual correction with hemiepiphysiodesis were predictive factors for the recurrence after surgery in hypophosphatemic rickets patients.

Age less than 10 years at the time of surgery was a prognostic factor for the recurrence of the deformity. Similar to previous studies [3, 8], we also confirmed that surgical treatment during early childhood or before skeletal maturity increases the risk of recurrence. Due to the unfused growth plate, the growth of patients with the disease could contribute to deformity recurrence in the physis despite adequate pharmacological treatment [14]. The younger the patient undergoes treatment, the higher the growth plate activity. Consequently, many authors have suggested that deformity correction should only be performed after skeletal maturity [7, 9, 15–17].

Many previous studies demonstrated recurrence after coronal angular deformity with hemiephiphysiodesis [18–21]. Leveille et al. reported a rebound rate of up to 52% of patients with a hip-knee-ankle angle of more than 5° [19]. Ramazanov et al. also reported a rebound phenomenon in 56% of valgus deformities and 24% of varus deformities [21]. Although those studies were not focused on rickets patients, the result from our study also found that gradual correction with hemiepiphysiodesis was a predictive factor for recurrence in hypophosphatemic rickets patients. This could be explained by the fact that to employ hemiepiphysiodesis for correcting deformity, patients must have viable growth plates and enough remaining time before skeletal maturity. Due to the limited number of gradual correction cases in this study, the result may not be able to conclude that gradual correction with hemiepiphysiodesis was a risk factor for recurrent deformity after surgery in rickets patients. Further studies with larger population sizes can provide stronger and more reliable results. However, previous studies reported that gradual correction with hemiepiphysiodesis was commonly encountered with a “rebound phenomenon”, leading to the concept of “sleeper plate” [22–24]. Consequently, with long periods of time until skeletal maturity, thereby increasing the risk of rebound phenomenon. Surgeons should attentively monitor the recurrence after implant removal, especially in younger patients.

Biochemical profiles also played an essential role in recurrence. According to Gizard et al., the incidence of recurrence seemed to be lower in patients with good metabolic control of rickets [7]. Low serum phosphate levels can also determine the risk of recurrence. Choi et al. concluded that serum phosphate level < 2.5 mg/dL was a risk factor for the recurrence of deformity [25]. However, our study did not find any significant association between biochemical profiles and deformity recurrence. This may be due to the missing data on biochemical profiles in our patients that were unable to be sufficiently demonstrated in statistical analysis.

In our study, the subtype of hypophosphatemic rickets was not associated with the recurrence. The fact that each subtype of hypophosphatemic rickets is rare, leading to a lack of sufficient data for proper analysis. Most patients in our study were X-linked hypophosphatemia, with only a few autosomal dominant and tumor-induced types to compare. There were also no previous works of literature on this topic. Therefore, a study on the effect of a subtype of hypophosphatemic rickets on recurrence in a larger sample size should be further investigated.

This study is one of only a few that have set out to identify predictive factors of deformity recurrence in hypophosphatemic rickets. The strengths of this study were, firstly, the collection of several potential parameters and analytical methods. Moreover, the analyses were performed with Cox proportional hazard analyses and Kaplan–Meier failure estimation curves to determine the effect of each potential parameter on deformity recurrence.

Nonetheless, this study had a few limitations. The parameters of the subjects were not independent variables. Additionally, this study was conducted on a limited number of patients. Lastly, amounts of data needed to be included for some parameters, such as vitamin D levels. The first two limitations were corrected using Cox proportional hazard analyses to describe the effects of potential parameters on deformity recurrence, with the population increasing by using bone segments as subjects. The third limitation was corrected by excluding missing parameters, such as vitamin D levels, from further analyses.

## Conclusion

In summary, we identified an age < 10 years at the time of surgery as a predictive factor for recurrent deformities after surgical correction in hypophosphatemic rickets. Gradual correction with hemiepiphysiodesis may also be a potential factor affecting the recurrence. These factors should be reviewed in patients with hypophosphatemic rickets before a corrective osteotomy and during the follow-up period. Consideration should be given to minimizing the risks for recurrent deformities.

## Data Availability

The datasets used and/or analyzed during the current study are available from the corresponding author on reasonable request.
